# Biochemical studies in experimentally *Escherichia coli* infected broiler chicken supplemented with neem (*Azadirachta indica*) leaf extract

**DOI:** 10.14202/vetworld.2015.1340-1345

**Published:** 2015-11-24

**Authors:** Vikash Sharma, K. K. Jakhar, Vikas Nehra, Sarvan Kumar

**Affiliations:** Department of Veterinary Pathology, Lala Lajpat Rai University of Veterinary & Animal Sciences, Hisar, Haryana, India

**Keywords:** biochemical studies, chicken, experimentally *Escherichia coli* infected broiler, neem leaf extract

## Abstract

**Aim:**

An experimental study was conducted on 192-day-old broiler chicks for evaluating the effect of 10% neem leaf extract (NLE) supplementationon biochemical parameters in chickens experimentally infected with *Escherichia coli* O78 at 10^7^ CFU/0.5 ml at 7 days of age.

**Materials and Methods:**

The 192-day-old broiler chicks were procured. These chicks were divided into two groups (A and B) containing 96 birds each on the 1^st^ day. Diet of all the chicks of Group A was supplemented with 10%NLE in water, whereas chicks of Group B were given feed and water devoid of NLE supplementation throughout the experiment. After rearing for 1 week, chicks of both the groups (A and B) were again divided into two subgroups (Group A into A1 and A2 and Group B into B1 and B2) of 54 and 42 birds, respectively. At the age of 7 days all the chicks of groups A1 and B1 were injected with *E. coli* O78 at 10^7^ CFU/0.5 ml intraperitoneally. Blood samples were collected from six chicks from each group at day 0, 2, 4, 7, 14, 21, 28 days post-infection and serum was separated for biochemical studies.

**Results:**

There was a significant increase in serum alanine transaminase (ALT), aspartate transaminase (AST), lactate dehydrogenase (LDH) activities, globulin concentration and a decrease in total protein (TP), albumin concentrations, and alkaline phosphatase (ALP) activity in both the infected groups. However, the changes in biochemical values, i.e., ALT, AST, LDH, ALP, TP, albumin, and globulin wereof lower magnitude in NLE supplemented group suggesting hepatoprotective and cardioprotective effect of NLE.

**Conclusions:**

Fromthe present study, it is reasonable to conclude that significant increase in the value of ALT, AST, LDH, globulin, and significant decrease in the value of ALP, TP, and albumin was of lower magnitude in supplemented infected group (A1) as compared to non-supplemented infected group (B1) suggesting hepatoprotective and cardioprotective effect of NLE.

## Introduction

India is experiencing rapid growth in the poultry sector. Poultry population of India is 729.21 million with an annual growth rate of 12.39% according to the Livestock Census, 2012. Total egg production is around 74.75 billion numbers during 2013-14 and acapita availability of egg is around 61 eggs/year [1]. The poultry meat production is estimated to be 2.69 million metric tonnes [1]. Poultry meat production of Haryana in the year 2011-2012 was 324,000 tonn, and average yield rate ananimal was 1.50 kg [2]. In recent years, commercial farms have grown in size and the poultry has undergone dramatic improvements in growth, feed efficiency and production. However, a major problem affecting the growth of the poultry industry in India is the occurrence of disease outbreaks. Some regions have reported a dramatic increase in the incidence of infectious disease outbreaks during this time of rapid expansion. Amongst these infections, *Escherichia coli* infection is quite common and causes a large number of disease conditions such as pericarditis, perihepatitis, airsacculitis, peritonitis, salpingitis, panophthalmitis, omphalitis, cellulitis, colisepticemia, coligranuloma, and Swollen-Head syndrome [3]. Colibacillosis is one of the main causes of economic losses in the poultry industry worldwide [4,5]. Though *E. coli* is the commensal resident in the intestinal tract of poultry, it often turns pathogenic under certain adverse conditions such as poor ventilation, overcrowding, and immunosuppression [6].

In recent years, efforts have been directed to control infectious diseases by use of herbal medicine which have fewer side effects and are ecologically safe. Neem(*Azadirachta indica*) is well-known for its medicinal properties. *A. indica* is a tree in the mahogany family Maliaceae. Neem is the most useful traditional medicine as a source of many therapeutic agents in the Indian culture and grows well in the tropical and semi-tropical countries. It has been shown that neem leaves extract acts as a growth promoter [7], improve performance and hematological parameters [8] and immune response [9,10] in broilers. In indigenous system of medicine, every part of the neem tree, *viz*. bark, leaves, fruits, seeds and extractsare used as medicine. Its extracts have antiviral, antibacterial, antifungal, anthelmintic, anti-allergic, anti-dermatic, and anti-inflammatory properties [11]. Neem oil extracted from its seeds is used in medicines, pest control and cosmetics, etc. Its leaves are used in the treatment of chicken pox. Neem also has anti-coccidial effect in broilers and is used as pesticide [12,13]. Neem leaves like most tropical tree leaves contain bioactive compounds [14,15] which may affect nutrient utilization. These bioactive compounds may also alter the hematological and serum biochemical parameters of animals. Unfortunately, the high fiber content in the neem leaf meal poses serious intake and digestibility problems in poultry diets [16]. Therefore, keeping in view the above facts, the present study was undertaken to study the biochemical changes in experimentally *E. coli* infected broiler chicken supplemented with neem (*A. indica*) leaf extract.

## Materials and Methods

### Ethical approval

The study was conducted after the approval of the Institutional Animal Ethics Committee.

### Preparation of neem leaf extracts (NLE)

Neem leaves collected from campus of CCS Haryana Agricultural University were shade dried and powdered. The aqueous extract was then prepared from powdered neem leaves. 100 g neem leaves powder was boiled in 1 L of water for 15 min and the extract obtained after straining was added to drinking water to make the volume of 1 L [11].

### Study design

An experiment was conducted to study the effect of NLE on clinicopathological and immune response of *E. coli* infected broiler chicks. For that 192-day-old broiler chicks were procured. The chicks were reared in the departmental animal house under strict hygienic conditions and were given feed and water *ad libitum*. These chicks were divided into two groups (A and B) containing 96 birds each. Diet of all the chicks of Group A was supplemented with 10% NLE in water whereas; chicks ofGroup B were given feed and water devoid of NLE supplementation throughout the experiment. After rearing for 1 week chicks of both the groups (A and B) were divided into two subgroups (Group A into A1 and A2 and Group B into B1 and B2) of 54 and 42 birds, respectively. At the age of 7 days, all the chicks of Groups A1 and B1 were injected with *E. coli* O78 at 10^7^ CFU/0.5 ml intraperitoneally.

### Collection of sample

Blood samples were collected from six chicks from each group at day 0, 2, 4, 7, 14, 21, 28 days post-infection and serum was separated for biochemical studies.

### Serum biochemical examination

Serum biochemical parameters were determined using Technicon Ames RA-50 chemistry analyzerusing diagnostic kits of Bayer. The levels of the following plasma constituents were determined: Total protein (TP), albumin, globulin and enzyme activities of alkaline phosphatase (ALP), lactate dehydrogenase (LDH), alanine transaminase (ALT), and aspartate transaminase (AST).

### Statistical analysis

The data for various serum biochemical parameters were subjected to statistical analysis using analysis of variance technique through *post-hoc*-DuncanLSDAlpha (0.05) using SPSS 16.0 version software. Standard errors of means were used to interpret the results [17].

## Results

### Serum AST

**Figure-1:**
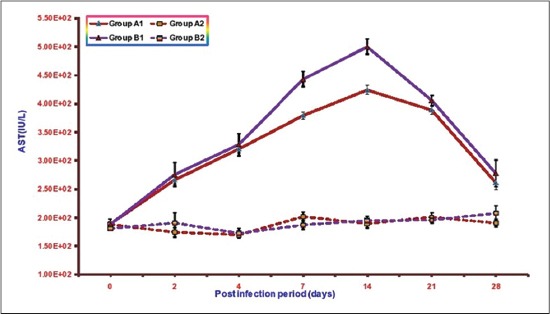
Mean serum asparate transaminase activities (IU/L) of broiler chicks in different experimental groups at different intervals.

The mean serum AST activities of different groups are illustrated in Figure-1. The mean AST activities in groups A1, B1, A2, and B2 ranges from 188.83±3.91 to 424.55±8.46, 188.27±3.48 to 500.5±13.14, 169.77±5.15 to 201.68±7.79, and 181.05±3.73 to 208.1±12.54, respectively. A significant increase (p<0.05) in serum AST was observed in both the infected Groups A1 and B1 as compared to non-infected Groups A2 and B2 from 2 DPI onward. However, this increase was lower in Group A1 in comparison to Group B1 and is significant at 7 DPI. The highest value of mean serum AST activity observed in Group B1 was 500.5±13.14 at 14 DPI and in Group A1 was 424.55±8.46 at 14 DPI. Between the non-infected groups, the serum AST values were almost similar.

### Serum ALT

**Figure-2:**
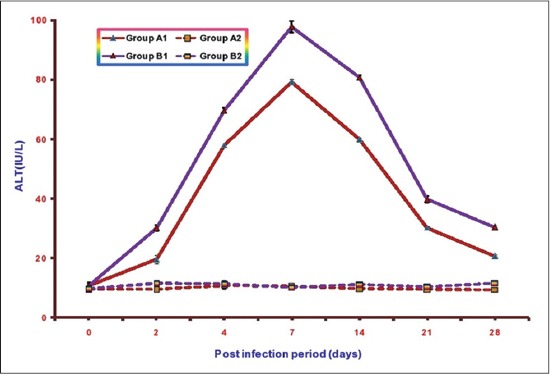
Mean serum alanine transaminase activities (IU/L) of broiler chicks in different experimental groups at different intervals.

The mean serumALT activities of different groups are illustrated in Figure-2. The mean ALT values in Groups A1, B1, A2, and B2 ranges from 10.73±0.78 to 79.31±0.94, 10.83±0.89 to 97.92±1.90, 9.38±0.50 to10.79±0.98 and 9.81±0.63 to 11.57±0.50, respectively. Mean serum ALT activities were significantly higher in both the infected groups as compared to non-infected groups from 2 DPI onward. However, the values of mean serum ALT were lower in Group A1 (infected group with NLE supplementation) as compared to Group B1 (infected group without NLE supplementation) from 2 DPI onward. The highest mean ALT activity observed in Group B1 was 97.92±1.90 at 7DPI and in Group A1 was 79.31±0.94 also at 7DPI. There was no significant difference in mean serum ALT values between non-infected Groups A2 and B2 except at 28 DPI where the value in Group B2 was significantly higher as compared to Group A2.

### TP

**Figure-3:**
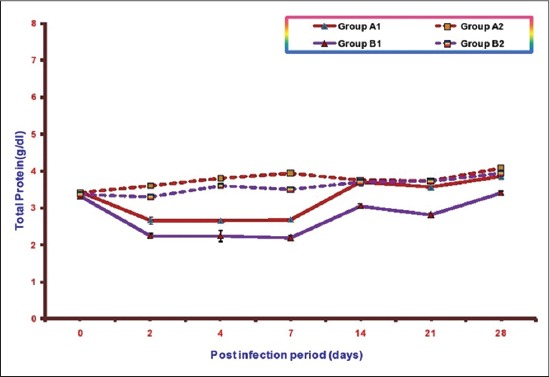
Mean total serum protein concentration activities (g/dl) of broiler chicks in different experimental groups at different intervals.

Mean total serum proteins (TSP) concentration of different groups at various intervals isillustrated in Figure-3. The mean TSP in Groups A1, B1, A2, and B2 ranges from 2.65±0.03 to 3.84±0.04, 2.19±0.07 to 3.40±0.04, 3.42±0.04 to4.06±0.04, and 3.30±0.04 to 3.94±0.04, respectively. A significant decrease in mean TSP concentrations was observed in both the infected groups as compared to control from 2 to 21 DPI. This decrease in NLE supplemented infected group (A1) was less in comparison to the infected group without NLE supplementation (B1). The lowest value of mean TSP observed in Group B1 was 2.19±0.07 at 7DPI and in Group A1 was 2.65±0.03 at 4DPI. There was no significant difference in serum values of TP between non-infected groups (A2 and B2) except at 2 and 7 DPI where it was significantly higher in Group A2.

### Albumin

**Figure-4:**
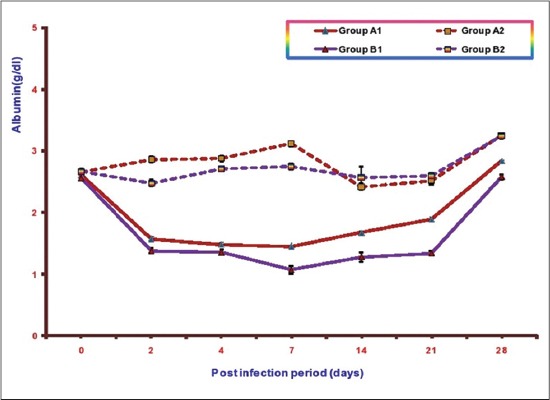
Mean serum albumin concentration (g/dl) of broiler chicks in different experimental groups at different intervals.

Mean serum albumin concentrations of different groups at various intervals are illustrated in Figure-4. The mean serum albumin concentrations in Groups A1, B1, A2, and B2 ranges from 1.45±0.02 to 284±0.03, 1.07±0.06 to 2.58±0.04, 2.42±0.05 to3.25±0.02, and 2.48±0.05 to 3.25±0.04, respectively. A significant decrease in mean serum albumin concentrations was observed in both the infected groups as compared to control from 2 DPI onward. This decrease was less in Group A1 (NLE supplemented infected group) in comparison to the infected Group B1 (without NLE supplementation). The lowest value of mean serum albumin concentration observed in Group B1 was 1.07±0.06 and in Group A1 was 1.45±0.02 at 7 DPI in both the groups. It was observed that there were higher mean serum albumin concentrations in A2 (NLE supplemented) as compared to B2 (without NLE supplementation) upto 7 DPI and no significant difference later on.

### Globulin

**Figure-5:**
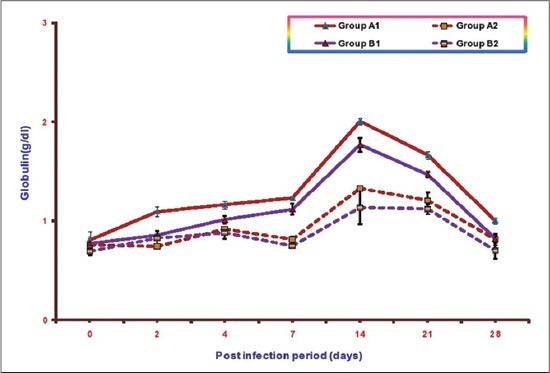
Mean serum globulin concentration (g/dl) of broiler chicks in different experimental groups at different intervals.

Mean serum globulin concentrations of different groups at various intervals areillustrated in Figure-5. The mean serum globulin concentrations in Groups A1, B1, A2, and B2 ranges from 0.81±0.08 to 2.01±0.03, 0.77±0.05 to 1.77±0.07, 0.76±0.01 to1.33±0.02, and 0.69±0.04 to 1.13±0.17, respectively. A significant increase in mean serum globulin concentrations was observed in both the infected groups as compared to control from 7 DPI onward. This increase was more in supplemented infected Group A1 (NLE supplemented infected group) in comparison to the infected Group B1 (without NLE supplementation). The highest value of mean serum globulin concentration observed in Group A1 was 2.01±0.03 at 14 DPI and in Group B1 was 1.77±0.07 also at 14 DPI. It was observed that there was no significant difference in mean serum globulin concentrations in between A2 (NLE supplemented) and B2 (without NLE supplementation).

### ALP

**Figure-6:**
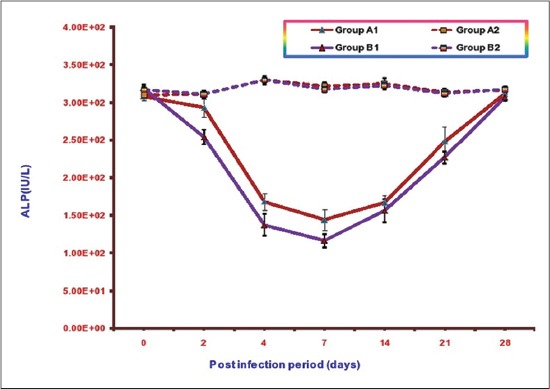
Mean serum alkaline phosphatase activities (IU/L) of broiler chicks in different experimental groups at different intervals.

Mean serum ALP concentrations of different groups at various intervals are illustrated in Figure-6. The mean serum ALP values in Groups A1, B1, A2, and B2 ranges from 144.77±14.08 to 311.75±4.79, 116.18±8.67 to 319.55±4.95, 310.28±3.14 to330.55±5.34, and 310.83±4.40 to 330.25±4.62, respectively. A significant decrease in mean serum ALPconcentrations was observed in both the infected groups as compared to control from 2 DPI to 21 DPI in B1 (without NLE supplementation) and 4 DPI to 21 DPI in A1(NLE supplemented infected group). This decrease was less in Group A1 (NLE supplemented infected group) in comparison to the infected Group B1 (without NLE supplementation). The lowest value of mean serum ALP observed in Group A1 was 144.77±14.08 and in Group B1 was 116.18±8.67 at 7DPI. There was no significant difference inmean serum ALP concentrations in A2 (NLE supplemented) andB2 (without NLE supplementation) throughout the experiment.

### LDH

**Figure-7:**
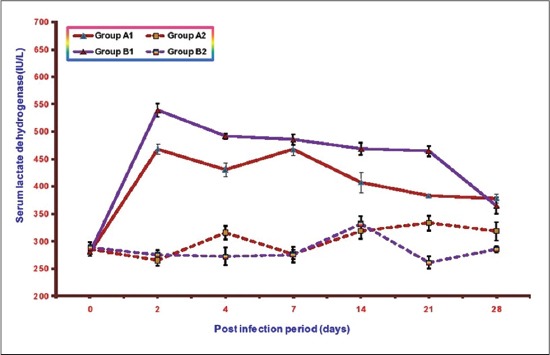
Mean serum lactate dehydrogenase activities (IU/L) of broiler chicks in different experimental groups at different intervals.

Mean serum LDHconcentrations of different groups at various intervals are illustrated in Figure-7. The mean serum LDH values in Groups A1, B1, A2 and B2 ranges from 280.52±4.12 to 468.32±9.72, 280.35±7.68 to 539.65±12.31, 265.45±10.47 to333.35±13.67, and 260.75±11.12 to 331.43±13.99, respectively. A significant increase in mean serum LDH concentrations was observed in both the infected groups as compared to control from 2 to 21 DPI. This increase was less in Group A1 (NLE supplemented infected group) in comparison to the infected Group B1 (without NLE supplementation). The highest value of mean serum ALP observed in Group A1 was 468.32±9.72 at 7 DPI and in Group B1 was 539.65±12.31at 2DPI.

## Discussion

Biochemical studies revealed a significant increase in the activities of serum AST and ALT, LDH in both the infected groups. Similar significant increase in serum AST and ALT activities have been reported by other workersin *E. coli* infection in broiler chickens [18-21]. Raheja and Jakhar, 2005 [22] reported significant increase in serum LDH activity in NLE supplemented fowl typhoid infected broiler chicken. The increase in serum AST is indicative of cellular injury to cardiac muscles and hepatocytes whereas elevated serum ALT is mostly due to hepatic injuries. The level of LDH increases in many processes in which there is cell necrosis such as hepatocellular necrosis, myocardial damage, renal necrosis, pancreatic necrosis, and hemolysis. The increase in these enzyme activities in NLE supplemented infected group (A2) was of significantly lower magnitude as compared to non-supplemented infected group (B1) throughout the experiment. These quantitative differences in the results of serum AST, ALT and LDH between the infected groups suggest hepatoprotective and cardioprotective effects of NLE supplementation. Similar effect of NLE was also observed by Raheja and Jakhar [22] in fowl typhoid infection and Saini [23] in *E. coli* infection.

Significant reduction in serum TP and albumin concentration was observed in both the infected groups. Decrease in these parameters in *E. coli* infection has been reported by other workers also [20-24]. According to Blood *et al*, [25], hypoproteinemia may be due to renal disease which lead to protein loss, liver damage which causes failure in plasma protein synthesis and congestive heart failure. The liver is a site for albumin synthesis. In the present study inappetence, damage to liver and kidney as evidenced by gross and histopathological studies were observed leading to decrease in TSP and albumin concentration. However, this decrease was significantly lower in NLE supplemented group indicating the hepatoprotective effect of NLE supplementation. Similar effect of NLE supplementation has been observed by Saini in *E. coli* infection [23] and by Raheja and Jakhar [22] in fowl typhoid infection in broiler chicken.

There was significant increase in the serum globulin value in both the infected groups. Hyperglobulinemia in *E. coli* infection has been reported by other workers also [23,26]. Hyperglobulinemia is associated with liver cirrhosis, hepatitis, Kuffer cell prolification.

Studies on ALP levels revealed that there was a significant decrease in serum ALP activity in both the infected groups but this decrease was more in the group with infection alone. In chicken serum, ALP was reduced in magnesium and zinc deficiency [27] thus indicating that birds infected with *E. coli* might be deficient of these minerals in the present study. Our observation is in accordance with other workers [23,28,29] they also reported decrease in serum ALP level in *E. coli* infection in poultry.

## Conclusion

Serum biochemical profiles of *E. coli* infected groups revealed that there were significant increase in serum ALT, AST, LDH activities, globulin concentration and decrease in TP, albumin concentrations, and ALP activity. These changes were of significantly lower magnitude in NLE supplemented group. Hence, it is reasonable to conclude from the present study that 10% NLE supplementation suggesting hepatoprotective and cardioprotective effect of NLE.

## Authors’ Contributions

VS and KKJ have designed the study and planned the research experiments. VS performed the research experiments. VN and SK helps in conducting experiment. All authors read and approved the final manuscript.
